# Plasma Glial Fibrillary Acidic Protein and Neurofilament Light Are Elevated in Bipolar Depression: Evidence for Neuroprogression and Astrogliosis

**DOI:** 10.1111/bdi.70029

**Published:** 2025-04-23

**Authors:** Matthew J. Y. Kang, Dhamidhu Eratne, Olivia Dean, Michael Berk, Adam J. Walker, Cassandra Wannan, Charles B. Malpas, Claudia Cicognola, Shorena Janelidze, Oskar Hansson, Jasleen Grewal, Philip B. Mitchell, Malcolm Hopwood, Christos Pantelis, Alexander F. Santillo, Dennis Velakoulis

**Affiliations:** ^1^ Neuropsychiatry Centre The Royal Melbourne Hospital Melbourne Victoria Australia; ^2^ Department of Psychiatry The University of Melbourne Parkville Victoria Australia; ^3^ Institute for Mental and Physical Health and Clinical Translation (IMPACT), School of Medicine Deakin University and Barwon Health Geelong Victoria Australia; ^4^ Florey Institute for Neuroscience and Mental Health Parkville Victoria Australia; ^5^ Orygen Parkville Victoria Australia; ^6^ Centre for Youth Mental Health The University of Melbourne Parkville Victoria Australia; ^7^ Melbourne School of Psychological Sciences The University of Melbourne Melbourne Victoria Australia; ^8^ Department of Medicine The Royal Melbourne Hospital Melbourne Victoria Australia; ^9^ Clinical Memory Research Unit, Department of Clinical Sciences Malmö, Faculty of Medicine Lund University Lund Sweden; ^10^ Memory Clinic Skåne University Hospital Malmö Sweden; ^11^ Alfred Mental and Addiction Health Alfred Health Melbourne Victoria Australia; ^12^ Discipline of Psychiatry and Mental Health, School of Clinical Medicine, Faculty of Medicine and Health University of New South Wales Sydney New South Wales Australia; ^13^ Professorial Psychiatry Unit Ramsay Clinic Albert Road Melbourne Victoria Australia; ^14^ Western Centre for Health Research & Education University of Melbourne & Western Health, Sunshine Hospital St Albans Victoria Australia; ^15^ Monash Institute of Pharmaceutical Sciences (MIPS) Monash University Melbourne Victoria Australia

**Keywords:** biological markers, bipolar disorder, depression, glial fibrillary acidic protein, mania, mental health, neurofilament light chain, psychiatry

## Abstract

**Background:**

Recent advances now allow detection of brain‐specific proteins in blood, including neurofilament light chain (NfL), a marker of axonal pathology, and glial fibrillary acidic protein (GFAP), indicative of astrocytic activation. Given the evidence of astroglial pathology and neuronal dysfunction in bipolar disorder, and ongoing debates on neuroprogression, we investigated plasma NfL and GFAP levels in affected individuals.

**Methods:**

This study analysed plasma NfL and GFAP measured in 216 individuals using Simoa. We used bootstrapped general linear models (GLM) to compare plasma NfL and GFAP levels between people with bipolar depression (*n* = 120) and healthy controls (*n* = 96), adjusting for age, sex, and weight. We examined associations between these biomarkers and clinical variables while adjusting for multiple comparisons. For sensitivity analyses, predictors were evaluated using Bayesian model averaging (BMA).

**Results:**

Plasma GFAP (*β* = 0.21 [0.07, 0.35], *p* = 0.006) and NfL (*β* = 0.06 [0.01, 0.10], *p* = 0.028) were elevated in people with bipolar depression. Illness duration was positively associated with NfL (*r* = 2.97, *p* = 0.002), and further supported by BMA analysis (posterior inclusion probability, PIP = 0.85). Age of onset was positively associated with GFAP (*r* = 0.246 *p* = 0.041), which was also supported by BMA analysis (PIP = 0.67).

**Conclusions:**

These findings indicate increased plasma NfL and GFAP levels in bipolar disorder. Our findings support the neuroprogression hypothesis, where prolonged illness duration contributes to neuroaxonal damage. Elevated GFAP in those with later onset suggests a role for neuroinflammation, potentially linked to increased cardiovascular and metabolic comorbidities.

## 1 Introduction

Bipolar disorder is a chronic psychiatric condition characterised by recurrent depressive and manic episodes, often accompanied by impairments in cognition and daily functioning that may persist even during remission. The observed association between bipolar disorder and long‐term cognitive impairment has led to the “neuroprogression” hypothesis [1, 2], which suggests that elevated levels of inflammation resulting from repeated mood episodes drive neuropathological processes that manifest as progressive cognitive decline, treatment resistance and structural brain changes [1, 3, 4]. However, the supporting research is mixed. For instance, an umbrella review by Carvalho et al. highlighted that much of the literature linking inflammation and bipolar disorder are limited by bias, fueling ongoing debates in the field [5, 6].

Blood‐based biomarker research investigating the pathophysiology of bipolar disorder has focused on systemic markers such as cytokines, which are not brain‐specific [7]. Recent technological advancements have made it possible to accurately measure picograms of brain‐derived proteins in the blood [8]. Two markers have drawn attention in the neurodegenerative disease space. Neurofilament light chain (NfL) is a cytoskeletal intermediate filament protein expressed in neurons, with elevated concentrations in blood following neuronal injury and degeneration [9, 10]. Glial fibrillary acidic protein (GFAP) is a component of the astrocyte cytoskeleton, with elevated concentrations being associated with astrocyte activation, a marker of neuroinflammation [11]. These proteins are of particular interest in bipolar disorder, given that the condition has been linked with neuronal dysfunction and neuroinflammation [3]. By examining these brain‐specific biomarkers, we can gain valuable insights into the neurobiological mechanisms underlying bipolar disorder.

Our recent systematic review [12] found that there was mixed and limited literature as to whether NfL levels are elevated in bipolar disorder compared to healthy controls. The mood states of these cohorts at the time of blood sampling were varied (euthymic, depressed, or mixed). Two studies specifically explored bipolar depression and found that plasma NfL was mildly elevated compared to healthy controls [13, 14]. Only one study investigated GFAP in a small number of people with bipolar disorder found that serum GFAP levels were similar to healthy controls [11]. None of these studies examined the correlation between plasma NfL/GFAP and clinical variables such as symptom severity.

Investigating NfL and GFAP in the blood of individuals with bipolar depression could provide valuable insights into the extent of neuronal pathology and astrocyte activation associated with the disorder, providing new avenues for therapeutic strategies. In this study, we aimed to further investigate the bipolar depression group by analysing plasma GFAP concentrations and comparing them with healthy controls. Furthermore, we aimed to build on our previous finding that NfL was higher in people with bipolar disorder [14], by exploring the relationships between NfL and GFAP with clinical and demographic factors in bipolar disorder to better understand what illness‐related factors are associated with higher biomarker levels.

## 2 Methods

### 2.1 Study Cohorts

This study, which is part of The Markers in Neuropsychiatric Disorders Study (The MiND Study, https://themindstudy.org), followed the Strengthening the Reporting of Observational Studies in Epidemiology (STROBE) reporting guidelines [15], and included 210 individuals from two multicentre cohorts. The bipolar depression cohort were from a registered clinical trial (ACTRN12612000830897), of adjunctive mitochondrial agents and N‐acetylcysteine [16]. Participants were at least 18 years old, met DSM‐IV‐TR diagnostic criteria for bipolar disorder, experiencing a bipolar depressive episode of at least moderate severity at the time of the study blood collection, and recruited from both inpatient and outpatient psychiatric services in Australia between 2013 and 2015. Participants were required to remain on stable treatment for at least 1 month prior to the study.

The healthy controls were pooled from the Cooperative Research Centre (CRC) for Mental Health Study [17], had no current or past psychiatric or neurological illness, and recruited from the general community in Australia between 2012 and 2018. We have reported plasma NfL values for this cohort previously [18].

### 2.2 Ethics

The authors assert that all procedures contributing to this work comply with the ethical standards of the relevant national and institutional committees on human experimentation and with the Helsinki Declaration of 1975, as revised in 2013. All procedures involving human subjects/patients were approved by the relevant Human Research Ethics Committees and all participants provided written informed consent prior to participation. This study was approved by the Melbourne Health Human Research Ethics Committee (MH HREC 2020.142).

### 2.3 Sample Analysis

The samples from both cohorts [16, 17] were collected at fasting state in plasma EDTA tubes, and stored at −80°C. Samples were randomised before analysis, and analyses were blinded to diagnosis. All plasma NfL levels were measured on the Quanterix SR‐X analyser using the same version of the Simoa NF‐Light kits. All plasma GFAP levels were measured using the same version of the Simoa GFAP Discovery kits. Both NfL and GFAP are suitable for retrospective analysis, as it is robust to multiple freeze–thaw cycles and time [19, 20, 21]. All analyses were completed in the same batch.

### 2.4 Statistical Analysis

All statistical analyses were performed using *R* (Version 4.2.2) Jamovi (Version 2.6.22.0) and GAMLj package (Version 3.4.2) [22, 23, 24]. To compare the demographic variables between bipolar depression and healthy controls, we used Mann–Whitney *U*‐tests and Pearson's chi‐square tests of independence.

General linear models (GLM) were used to compare NfL and GFAP levels between healthy controls and individuals with bipolar disorder. NfL and GFAP values were entered as dependent variables and were log_10_ transformed due to positively skewed distributions, and we reported raw values for easier interpretation. Cohort group, age, sex and weight were entered as independent variables, and 95% confidence intervals were computed (nonparametric bootstrapping, 1000 replicates), with statistical significance defined as any confidence interval not including the null (at 95% level).

#### 2.4.1 Exploratory Analysis of Clinicodemographic Variables and Biomarker Values

##### 2.4.1.1 Conventional Frequentist Analysis

For exploratory analysis of clinical and demographic factors that may explain the elevated biomarker level in bipolar disorder, we used Spearman's rho for continuous variables and Welch's *t*‐test for categorical variables to analyses their associations. We adjusted for multiple comparisons using Benjamini‐Hochberg false discovery rate (FDR) correction. All statistical assumptions were checked, including multicollinearity in linear models using the variance inflation factor (VIF).

##### 2.4.1.2 Bayesian Model Averaging

To further mitigate the problem of using multiple predictors, we used Bayesian model averaging (BMA) a validated statistical technique that allows for all potential predictors to be examined in the model in parallel, while simultaneously considering all possible formulations of the model [25]. The overall statistical solution is composed of all possible combinations of predictors weighted by model fit. As the best‐fitting combinations contribute more to the final model, and the worst‐fitting models contribute less, this naturally deals with the problem of model selection without having to impose model selection bias.

Briefly, for *p* predictors, BMA estimates all possible models corresponding to the 2^*p*^ possible combinations of predictors. The fit of each model was then evaluated using the log of the posterior odds. Parameter estimates were averaged over all models, weighted for the fit of each model, which inherently adjusted for the uncertainty associated with model selection bias.

The importance of each predictor was evaluated by the posterior inclusion probability (PIP) value, which was the probability that the parameter is not zero given the data. Predictors with PIP values > 0.5 were considered ‘important’. Predictions of NfL or GFAP for each patient were computed using the posterior predictive distribution averaged across all models. BMA was performed using the Bayesian adaptive sampling package [26]. As one variable (number of depressive episodes) had 11 missing values, all BMA analyses were run with and without this variable to ensure that the results were consistent.

## 3 Results

### 3.1 Study Cohort Characteristics

A total of 216 participants had adequate plasma samples available for NfL and GFAP analyses. The 120 participants with bipolar disorder comprised of 75 (62%) females and 45 (38%) males, with a mean ± SD age of 43.9 ± 12.0 years and weight of 84.3 ± 21.0 kg. For healthy controls (*n* = 96), there were 50 (52%) females and 46 (48%) males, with a mean ± SD age of 44.7 ± 14.4 years and weight of 77.3 ± 15.4 kg. The bipolar depression cohort and the healthy controls were not statistically different in terms of their demographic measures, including their weight (Table 1).

**TABLE 1:** Descriptive comparison of the cohorts.

	Bipolar depression (*n* = 120)	Controls (*n* = 96)
Gender		
Female	75 (62%)	50 (52%)
Male	45 (38%)	46 (48%)
Age (years)	43.9 (12.0)	44.7 (14.4)
Weight (kg)	84.3 (21.0)	77.3 (15.4) [*n* = 87]
Height (cm)	170.3 (9.1) [*n* = 119]	170.3 (10.8) [*n* = 89]
NfL (pg/mL)	8.7 (12.8)	9.4 (14.2)
GFAP (pg/mL)	95.55 (49.31)	75.47 (46.94)

*Note:* Values expressed as mean (SD); *n* (%).

Abbreviations: GFAP = glial fibrillary acidic protein; NfL = Neurofilament light chain.

As for the clinical characteristics of the bipolar depression cohort, their mean age at diagnosis by a health clinician was 34 ± 11 years old, duration of bipolar illness (from age of self‐reported onset of symptoms) was 23 ± 11 years, 78 (65%) individuals were prescribed at least one mood stabiliser, and 75 (69%) individuals reported having experienced 20+ depressive episodes in their lifetime. With regards to their symptom severity and functioning, their mean MADRS was 29.2 ± 5.2, BDRS was 25.7 ± 6.6, HAM‐A was 17.6 ± 5.7, YMRS was 3.9 ± 3.4, Q‐LES‐Q was 40.1 ± 12.7 and CGI‐S was 4.7 ± 0.8.

### 3.2 Biomarker Levels in Bipolar Depression and Healthy Controls

The raw mean ± SD of plasma NfL was 8.7 ± 12.8 pg/mL in bipolar depression and 9.4 ± 14.2 pg/mL in healthy controls. The raw mean ± SD of GFAP was 95.6 ± 49.3 pg/mL in bipolar depression and 75.5 ± 46.9 pg/mL in healthy controls. Plasma GFAP was significantly elevated in people with bipolar depression compared to healthy controls after adjusting for age, sex and weight (*β* = 0.21 [0.07, 0.35], *p* = 0.006). Consistent with our previous finding [14], log‐transformed NfL was also significantly elevated in people with bipolar depression compared to healthy controls (*β* = 0.06 [0.01, 0.10], *p* = 0.028; Figure S1).

### 3.3 Clinical Correlates of NfL and GFAP

Age positively correlated with both NfL and GFAP (Table 2). People who had a history of substance use disorder (SUD) in their lifetime had significantly lower GFAP concentration than those without (*t* = 2.97, df = 95.75, adjusted *p* = 0.024). However, people with a SUD history were younger than those without (mean 38.2 ± 8.6 years vs. 46.4 ± 12.4 years; *F* = 13.63, *p* < 0.001). The multivariable GLM including age and SUD history found that SUD was not significantly associated with GFAP (*β* = −0.33; [−0.68, 0.02]; *p* = 0.058), suggesting therefore, that the difference was attributable to age.

**TABLE 2:** Clinical correlates of NfL and GFAP in bipolar depression.

Characteristic	*N* = 120, mean (SD); *n* (%)	NfL correlation (*p*‐value)	GFAP correlation (*p*‐value)
Age (years)	43.89 (11.95)	**0.468 (*p* < 0.001)**	**0.34 (*p* = 0.002)**
Gender	Females: 75 (63%) Male: 45 (37%)	0.281 (*p* = 0.822)	0.13 (*p* = 0.362)
Weight (kg)	84.3 (21.0)	−0.162 (*p* = 0.196)	−0.113 (*p* = 0.389)
History of substance dependence	37 (31%)	2.265 (*p* = 0.097)	**2.970 (*p* = 0.029)**
Duration of Illness (*n* = 117)	23 (11)	**0.331 (*p* = 0.002)**	0.15 (*p* = 0.271)
Age at diagnosis (*n* = 119)	34 (11)	**0.253 (*p* = 0.026)**	**0.246 (*p* = 0.040)**
Mood stabiliser	78 (65%)	−1.417 (*p* = 0.336)	−2.117 (*p* = 0.141)
Lithium	18 (18%)	0.386 (*p* = 0.822)	−0.647 (*p* = 0.729)
Antipsychotic	72 (60%)	1.034 (*p* = 0.527)	0.116 (*p* = 0.908)
No. of depressive episodes (*n* = 109)		0.185 (*p* = 0.173)	0.127 (*p* = 0.33)
1–10	15 (14%)		
11–20	19 (17%)		
20+	75 (69%)		
MADRS	29.2 (5.2)	−0.03 (*p* = 0.822)	0.114 (*p* = 0.389)
BDRS	25.7 (6.6)	−0.073 (*p* = 0.580)	0.023 (*p* = 0.908)
HAM‐A	17.6 (5.7)	−0.084 (*p* = 0.572)	−0.012 (*p* = 0.908)
YMRS	3.9 (3.4)	−0.078 (*p* = 0.580)	−0.158 (*p* = 0.271)
Q‐LES‐Q	40.1 (12.7)	0.14 (*p* = 0.301)	0.083 (*p* = 0.566)
CGI‐S	4.7 (0.8)	0.041 (*p* = 0.822)	0.149 (*p* = 0.271)
IL‐6	1.90 (1.53)	0.099 (*p* = 0.527)	−0.031 (*p* = 0.892)
TAC (*n* = 119)	0.47 (0.11)	−0.005 (*p* = 0.958)	0.102 (*p* = 0.447)
GFAP	95.55 (49.31)	**0.455 (*p* < 0.001)**	N/A
NfL	8.68 (12.76)	N/A	**0.455 (*p* < 0.001)**

*Note: p*‐values corrected for multiple comparisons; correlation with Spearman's rho for continuous variables and Welch's *t*‐test for categorical variables; bolded indicating *p* < 0.05.

Abbreviations: BDRS = Bipolar Depression Rating Scale; CGI‐S = Clinical Global Impressions—Severity Scale; GFAP = glial fibrillary acidic protein; HAM‐A = Hamilton Anxiety Rating Scale; IL‐6 = Interleukin‐6; MADRS = Montgomery–Åsberg Depression Rating Scale; NfL = Neurofilament light chain; Q‐LES‐Q = Quality of Life Enjoyment and Satisfaction Questionnaire; TAC = Total Antioxidant Capacity; YMRS = Young Mania Rating Scale.

**FIGURE 1:**
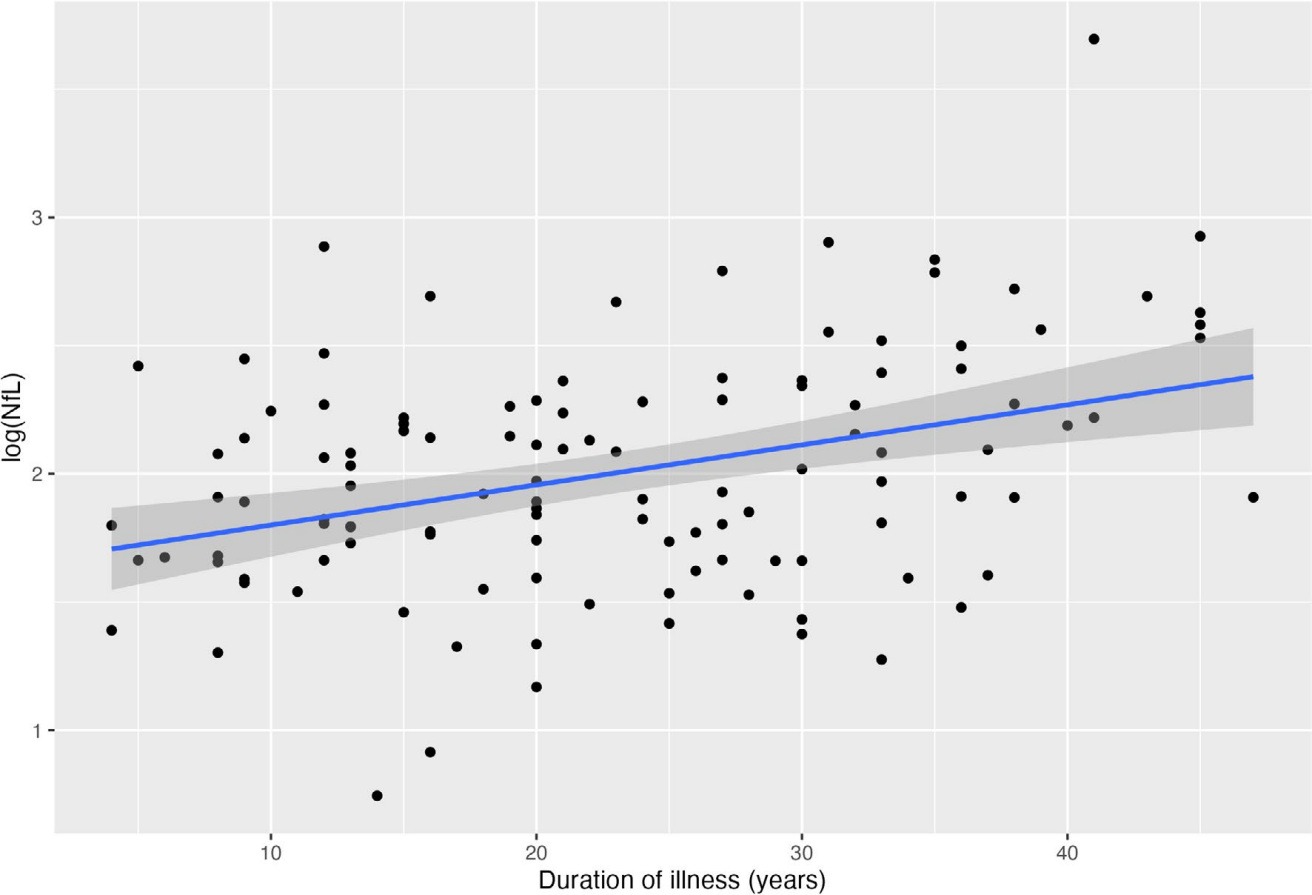
Scatterplot of plasma NfL and duration of illness.

**FIGURE 2:**
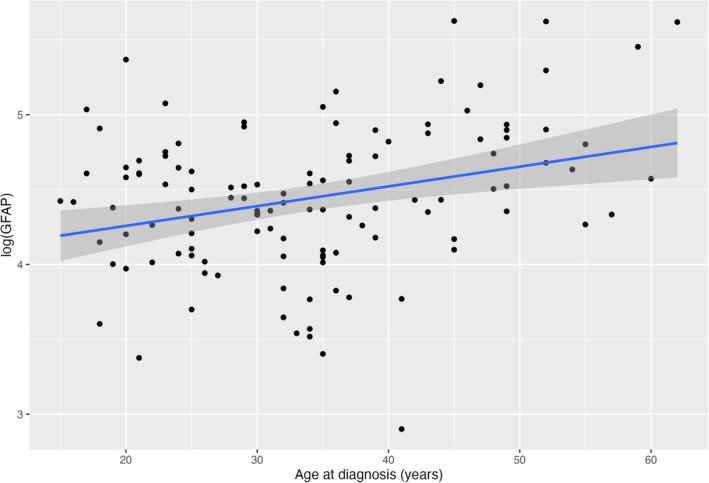
Scatterplot of plasma GFAP and age at diagnosis.

Both longer duration of illness and older age at diagnosis were positively correlated with higher NfL concentration (Figure 1). Older age at diagnosis positively correlated with higher GFAP concentration (Figure 2). Duration of illness (VIF = 1.69) and age at diagnosis (VIF = 2.08) were not significant for multicollinearity (VIF < 3.0). Neither duration of illness nor age at diagnosis were significant in the GLMs when they were added with age to predict NfL or GFAP.

### 3.4 BMA Analysis

#### 3.4.1 Plasma NfL

**FIGURE 3:**
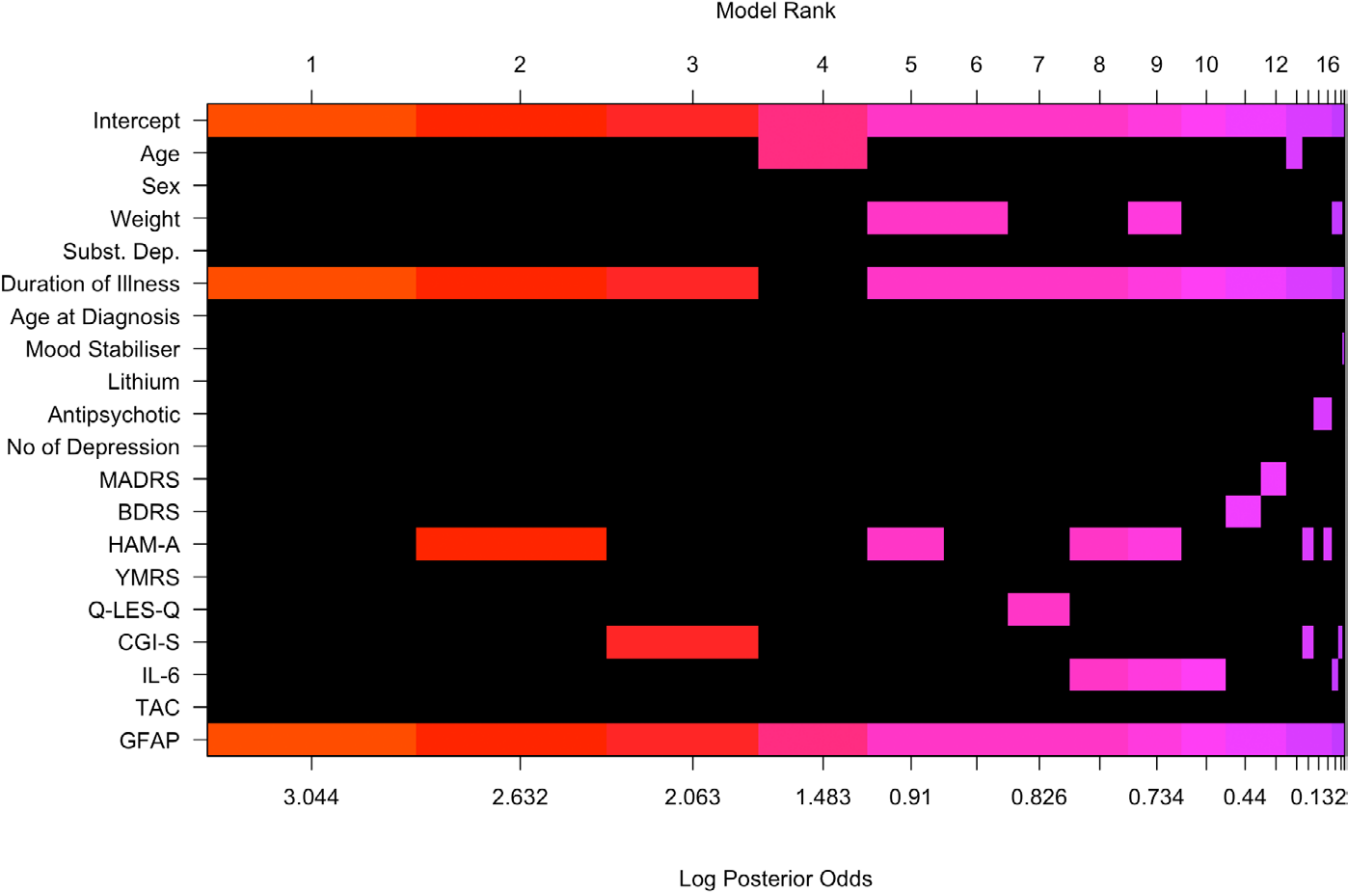
Visualisation of the model space from the BMA analysis of NfL correlates. BDRS = Bipolar Depression Rating Scale; CGI‐S = Clinical Global Impressions—Severity Scale; GFAP = glial fibrillary acidic protein; HAM‐A = Hamilton Anxiety Rating Scale; IL‐6 = Interleukin‐6; MADRS = Montgomery–Åsberg Depression Rating Scale; NfL = Neurofilament light chain; Q‐LES‐Q = Quality of Life Enjoyment and Satisfaction Questionnaire; TAC = Total Antioxidant Capacity; YMRS = Young Mania Rating Scale.

BMA revealed several important predictors of NfL. The posterior inclusion probabilities (PIP) are shown in Table 3. The strongest evidence was observed for GFAP (PIP = 1), whilst duration of illness was also strongly supported (PIP = 0.85). The remaining predictors (Table 3), including age, had PIPs < 0.5, and only GFAP was a significant independent predictor of NfL, with its 95% CI not capturing zero. Taken in the context of the PIP value > 0.5, this indicates that GFAP is important to include in any well‐fitting model predicting NfL using the possible variables, whilst there is some doubt about the magnitude of the other predictors in predicting NfL. A visual representation of the importance of each predictor in the top 20 models is shown in Figure 3, and the predictors ranked by their PIP in Figure S2.

**TABLE 3:** BMA analysis for prediction of plasma NfL and GFAP concentrations.

Predictor	NfL		GFAP	
	B [95% CI]	PIP	B [95% CI]	PIP
Age	0.13 [0–1.22]	0.26	0.13 [0–1.22]	0.16
Sex	0.51 [−0.51–6.27]	0.11	0.51 [−0.51–6.27]	0.06
Weight	0 [0–0]	0.47	0 [0–0]	0.04
Substance dependence history	−0.93 [−11.03–0.1]	0.07	−0.93 [−11.03–0.1]	0.08
Duration of illness	−0.05 [−0.58–0]	0.85	−0.05 [−0.58–0]	0.08
Age at diagnosis	0.78 [0–1.74]	0.09	0.78 [0–1.74]	0.67
Mood stabiliser	1.67 [−0.02–17.29]	0.07	1.67 [−0.02–17.29]	0.13
Lithium	0.32 [0–0]	0.10	0.32 [0–0]	0.05
Antipsychotic	0.32 [−0.39–2.65]	0.15	0.32 [−0.39–2.65]	0.05
MADRS	0.04 [0–0.58]	0.09	0.04 [0–0.58]	0.07
BDRS	0 [−0.07–0]	0.09	0 [−0.07–0]	0.05
HAM‐A	0.1 [0–1.22]	0.32	0.1 [0–1.22]	0.10
YMRS	−0.23 [−2.44–0]	0.07	−0.23 [−2.44–0]	0.11
Q‐LES‐Q	0 [0–0]	0.11	0 [0–0]	0.04
CGI‐S	1.29 [0–11.92]	0.15	1.29 [0–11.92]	0.14
IL‐6	−0.14 [−1.57–0]	0.26	−0.14 [−1.57–0]	0.07
TAC	5.86 [0–65.95]	0.07	5.86 [0–65.95]	0.11
GFAP	4.14 [2.24–6.05]	1.00	N/A	N/A
NfL	N/A	N/A	4.14 [2.24–6.05]	1.00

Abbreviations: 95% CI = 95% credible intervals; B = model coefficients computed as the mean of the posterior distribution; PIP = posterior inclusion probability; SE = standard deviation of the posterior distributions.

#### 3.4.2 Plasma GFAP

**FIGURE 4:**
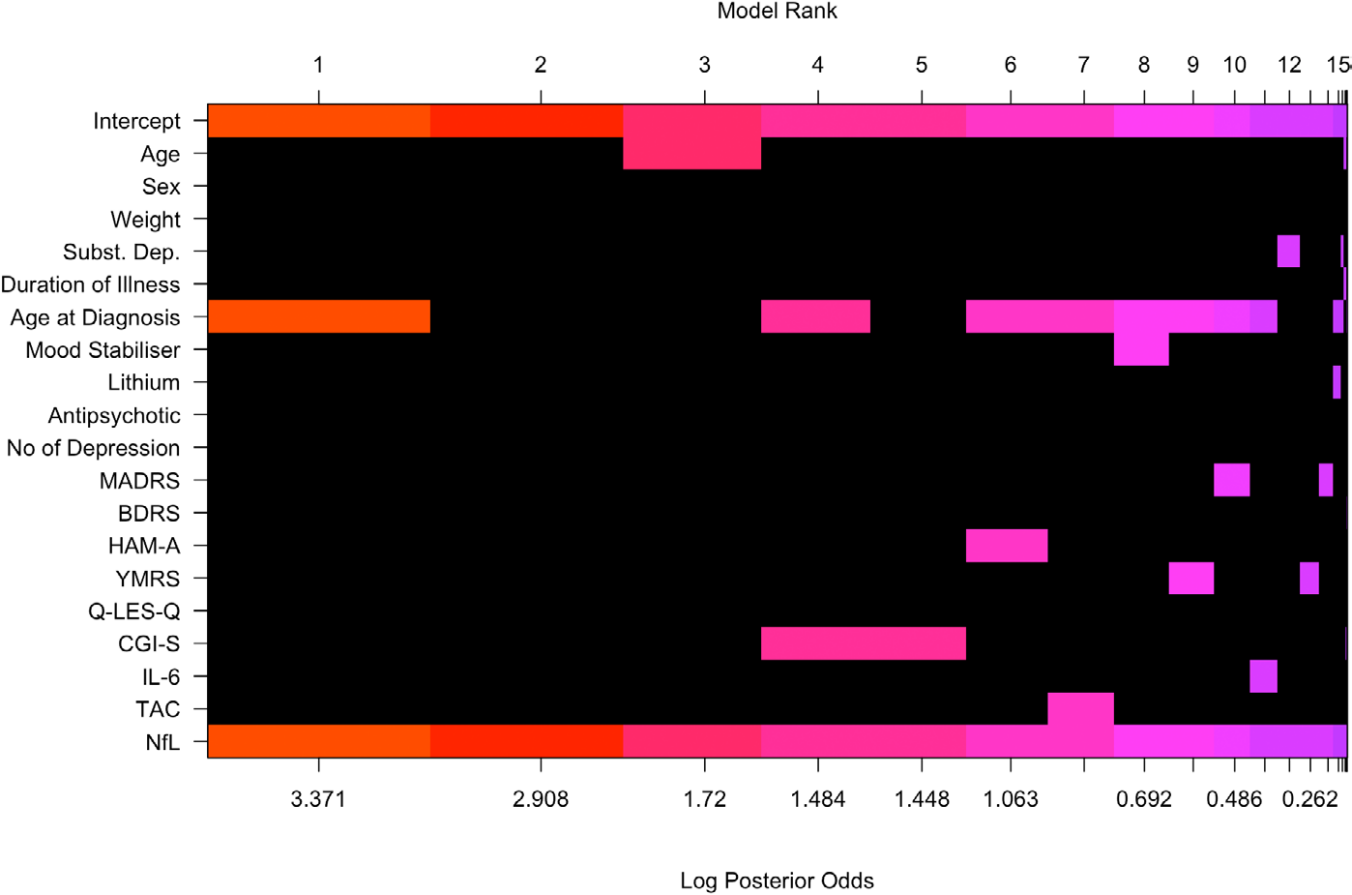
Visualisation of the model space from the BMA analysis of GFAP correlates. BDRS = Bipolar Depression Rating Scale; CGI‐S = Clinical Global Impressions—Severity Scale; GFAP = glial fibrillary acidic protein; HAM‐A = Hamilton Anxiety Rating Scale; IL‐6 = Interleukin‐6; MADRS = Montgomery–Åsberg Depression Rating Scale; NfL = Neurofilament light chain; Q‐LES‐Q = Quality of Life Enjoyment and Satisfaction Questionnaire; TAC = Total Antioxidant Capacity; YMRS = Young Mania Rating Scale.

NfL (PIP = 1) was considered an important predictor (Figure 3; Table 3), whilst age at diagnosis was a moderately strong predictor (PIP = 0.67) but its estimate was less precise and included 0 in its 95% CI (B = 0.78, 95% CI: 0–1.74), which taken together implies that this predictor is important to include in any well‐fitting model, but there is considerable uncertainty regarding the specific magnitude of this relationship. The other variables, including age, were not considered to be strong predictors of GFAP. A visual representation of the importance of each predictor in the top 20 models is shown in Figure 4, and the predictors ranked by their PIP in Figure S3.

## 4 Discussion

This is the largest study to date exploring the relationship between brain‐specific markers of neurodegeneration and astroglial dysfunction in people with bipolar depression. By extending on our previous work [14], we found that both plasma NfL and GFAP are elevated in people with bipolar depression compared to healthy controls. Plasma NfL was associated with a longer duration of illness and may represent a marker of illness stage. Our novel finding that plasma GFAP was elevated in bipolar depression suggests that there is increased activation of astrocytes, which has been linked with neuroinflammation, adding to the debated literature about the role of inflammation in bipolar disorder [27, 28, 29] and the neuroprogression hypothesis [5, 6]. Plasma GFAP was also positively associated with a later age of bipolar diagnosis, which suggests that those with a later age of onset have a distinct neurobiological process. These proteins could serve as objective biomarkers that identify homogenous phenotypes in bipolar disorder, and address the problems of heterogeneity which hinder research efforts [30].

The difference in NfL concentrations between people with bipolar disorder and unaffected individuals was small and only evident after adjusting for known covariates. This contrasts with the markedly elevated concentrations (at least two‐fold) seen in neurodegenerative disorders such as dementia and multiple sclerosis [31, 32], suggesting that the subtle increase in bipolar disorder may be due to a different or less intense process. Aggio et al.'s study [13] similarly found that NfL was mildly elevated in people with bipolar depression (*n* = 45) and linked this to neuronal distress with axonal damage due to an ageing‐related process, or “accelerated brain ageing.” Accelerated brain ageing refers to the age gap between chronological and the estimated neuroanatomical age, which has been observed in various psychiatric disorders including bipolar disorder [33, 34]. Plasma NfL has been proposed as a potential marker of brain ageing [35, 36] due to its age‐related increases [37]. As the brain atrophies with age [38], NfL is released from dendrites and axons into CSF and blood [39]. The subtle elevation of NfL levels in bipolar disorder after adjustment for chronological age supports the notion that there is accelerated ageing in bipolar disorder, adding to the current debate in the literature [33, 34, 40].

Moreover, our finding that plasma NfL levels correlate with the duration of bipolar disorder may be evidence of the progressive burden of the condition on the brain. This supports the “neuroprogression” hypothesis, which postulates that a subset of people have a progressive trajectory due to pathological rewiring and degeneration of the brain over the course of the illness [1, 41, 42]. Longitudinal neuroimaging studies have found that people with bipolar disorder have faster cortical atrophy [43] and ventricular enlargement [44] beyond what is expected with normal ageing [45]. Similarly, our BMA analysis also identified that duration of illness, and not chronological age, was better predictive of plasma NfL levels. This negative finding of chorological age not predicting plasma NfL was surprising given it is contrary to well‐established literature [37], including our own conventional frequentist analysis in this study. Considering the broader literature, our finding suggests that the “neuroprogressive” pathology of bipolar disorder outpaces chronological age‐related brain changes, and this has a neuroinflammatory component given the elevated GFAP levels. In clinical practice, NfL may have utility in identifying and monitoring individuals with bipolar disorder vulnerable to neuroprogression, similar to how it has been proposed as a biomarker of cognition and prognosis in neurological conditions including Parkinson's disease [46] and mild cognitive impairment [47].

This novel finding that the duration of bipolar disorder is a better predictor of NfL than chronological age highlights the strength of BMA analysis in being able to consider a broad range of possible predictors, examine a wide range of models based on all the variables available, and provide a value that represents the importance of each predictor across all possible models. This contrasts with the traditional frequentist approach, which can only consider one model at a time introducing bias [48]. Given that plasma NfL level is impacted by many factors [8, 18], not limited to the person's age, weight, and physical health, the ability of BMA to consider a wide range of predictors uncovered an interesting insight in this study. Nonetheless, the limited age range of our cohort warrants further research incorporating younger and older cohorts with bipolar disorder. In addition, studies investigating the three‐way relationship between NfL, neuroimaging, and the burden of bipolar disorder (longer duration, number of manic and/or depressive episodes) is required to validate this hypothesis that the duration of bipolar illness has a greater burden on the brain age than chronological age.

Our finding of elevated GFAP levels in bipolar disorder suggests that neuroinflammation may be a component of this condition, especially in those with later onset of illness. This may be connected to the increased prevalence of cardiovascular and metabolic conditions in older adults, which are known to cause neuroinflammatory changes and oxidative stress in the brain [49]. Although we did not collect details about their cardiovascular health, the Clinical and Health Outcomes Initiative in Comparative Effectiveness for Bipolar Disorder study (Bipolar CHOICE), which had a comparable cohort age (38.9 ± 12.1 years), found that individuals with a later age onset of bipolar disorder were more likely to have underlying medical comorbidities [50]. Combining this with our finding that GFAP was higher in those with later age of onset, this may represent an increased expression and release of GFAP from astrocytic activation as a form of defence response to neuroinflammation. However, GFAP did not correlate with peripheral interleukin‐6 (IL‐6) or total antioxidant capacity (TAC), suggesting that peripheral and CNS inflammation may contribute to the pathophysiology of bipolar disorder in distinct ways. Future studies should characterise cardiovascular and metabolic risk and compare this with biomarker levels.

It remains unclear whether mild increases in plasma NfL and/or GFAP in bipolar disorder are related to the trait (bipolar disorder) or state (depression, mania or mixed state) [10, 51]. Previous research by Steinacker et al. found that GFAP correlated with the severity of unipolar depressed state as measured by the MADRS [11]. We found that the severity of depressive symptoms did not correlate with plasma NfL nor GFAP suggests that unipolar and bipolar depression may have different underlying neurobiological processes. Despite lithium's postulated neuroprotective role [52], it also did not have a significant effect on plasma NfL nor GFAP, though it may have been underpowered considering that plasma NfL and GFAP levels were only mildly elevated in people with bipolar disorder. Furthermore, antipsychotic medication has been associated with elevated CSF NfL levels [53], but our study did not replicate this finding in plasma NfL.

This study has several limitations. The cross‐sectional nature of the study makes it difficult to make causal inferences, so future studies with serial samples and longitudinal clinical data are needed. We lacked other important clinical information about the people with bipolar disorder, including renal function, previous medications, cognition and number of manic episodes. Investigating these biomarkers in manic states may yield important insights, although recruiting participants in acute mania is challenging. Furthermore, detailed history including the type and quantity of substance use would have been useful to better understand the correlation between substance use disorder history and NfL/GFAP, especially given the emerging evidence that chronic cocaine use and alcoholism are associated with elevated NfL levels [54, 55], highlighting a potential area for future research. Additional research involving cohorts in different phases of bipolar disorder (i.e., index episode, acute manic episode) is needed to validate whether the differences in NfL and GFAP are generalisable to the disorder or are episode dependent. Finally, we recommend future research to use a multimodal approach, combining fluid‐based biomarker levels with neuroimaging to strengthen the findings.

In conclusion, this is the largest study to date that has investigated plasma NfL and GFAP in people with bipolar disorder. We found that both are mildly elevated compared to healthy controls, with higher NfL associated with a longer duration of bipolar disorder, while GFAP was associated with a later age of onset. Taken together, our study further supports the neuroprogression hypothesis, as individuals with bipolar disorder had elevated GFAP levels, a neuroinflammatory marker, compared to healthy controls, and those with longer duration of illness exhibited higher neuroaxonal burden outpacing chronological age. Moreover, these biomarkers may be potential candidates to identify different stages and phenotypes of bipolar disorder, such as neuroprogression or later onset of illness. Further studies with longitudinal data are needed to further validate our findings and better understand the neurobiological underpinnings of bipolar disorder.

## Author Contributions

Matthew J.Y. Kang: formulating research question, study design, data analysis, manuscript writing and submission. Dhamidhu Eratne: formulating research question, study design, data analysis, manuscript revision and supervision. Charles B. Malpas: formulating research question, study design, data analysis, statistical support and supervision. Alexander F. Santillo: formulating research question, study design, data analysis, statistical support and supervision. Cassandra Wannan: study design, manuscript revision. Claudia Cicognola: study design, manuscript revision. Shorena Janelidze: study design, data analysis, manuscription revision. Olivia Dean: study design, manuscript revision. Oskar Hansson: study design, manuscript revision. Michael Berk: study design, manuscript revision. Adam J. Walker: study design, manuscript revision. Jasleen Grewal: study design, manuscript revision. Dennis Velakoulis: study design, data analysis, manuscript revision and supervision. Philip B. Mitchell: study design, manuscript revision and supervision. Christos Pantelis: study design, manuscript revision and supervision. Malcolm Hopwood: study design, manuscript revision and supervision.

## Conflicts of Interest

O.H. has acquired research support (for the institution) from AVID Radiopharmaceuticals, Biogen, C2N Diagnostics, Eli Lilly, Eisai, Fujirebio, GE Healthcare, and Roche. In the past two years, he has received consultancy/speaker fees from Alzpath, BioArctic, Biogen, Bristol Meyer Squibb, Eisai, Eli Lilly, Fujirebio, Merck, Novartis, Novo Nordisk, Roche, Sanofi and Siemens. O.D. has received grant support from the Brain and Behaviour Foundation, Simons Autism Foundation, Stanley Medical Research Institute, Deakin University, Lilly, NHMRC and ASBDD/Servier. She has also received in kind support from BioMedica Nutracuticals, NutritionCare and Bioceuticals. All other authors report no biomedical financial interests or potential conflicts of interest.

## Supporting information


Figure S1.
Figure S2.
Figure S3.
Table S1.

## Data Availability

Deidentified and pooled data is available on request from the corresponding author.
