# Supplementary material for: Plasma Glial Fibrillary Acidic Protein and Neurofilament Light Are Elevated in Bipolar Depression: Evidence for Neuroprogression and Astrogliosis
Source: Bipolar Disord. 2025 Apr 23;27(5):379–88. doi: 10.1111/bdi.70029 (PMC12397446; doi:10.1111/bdi.70029)
Supplement: Supplementary file 1 — Figure S1. Figure S2. Figure S3. Table S1. [file BDI-27-379-s001.docx]

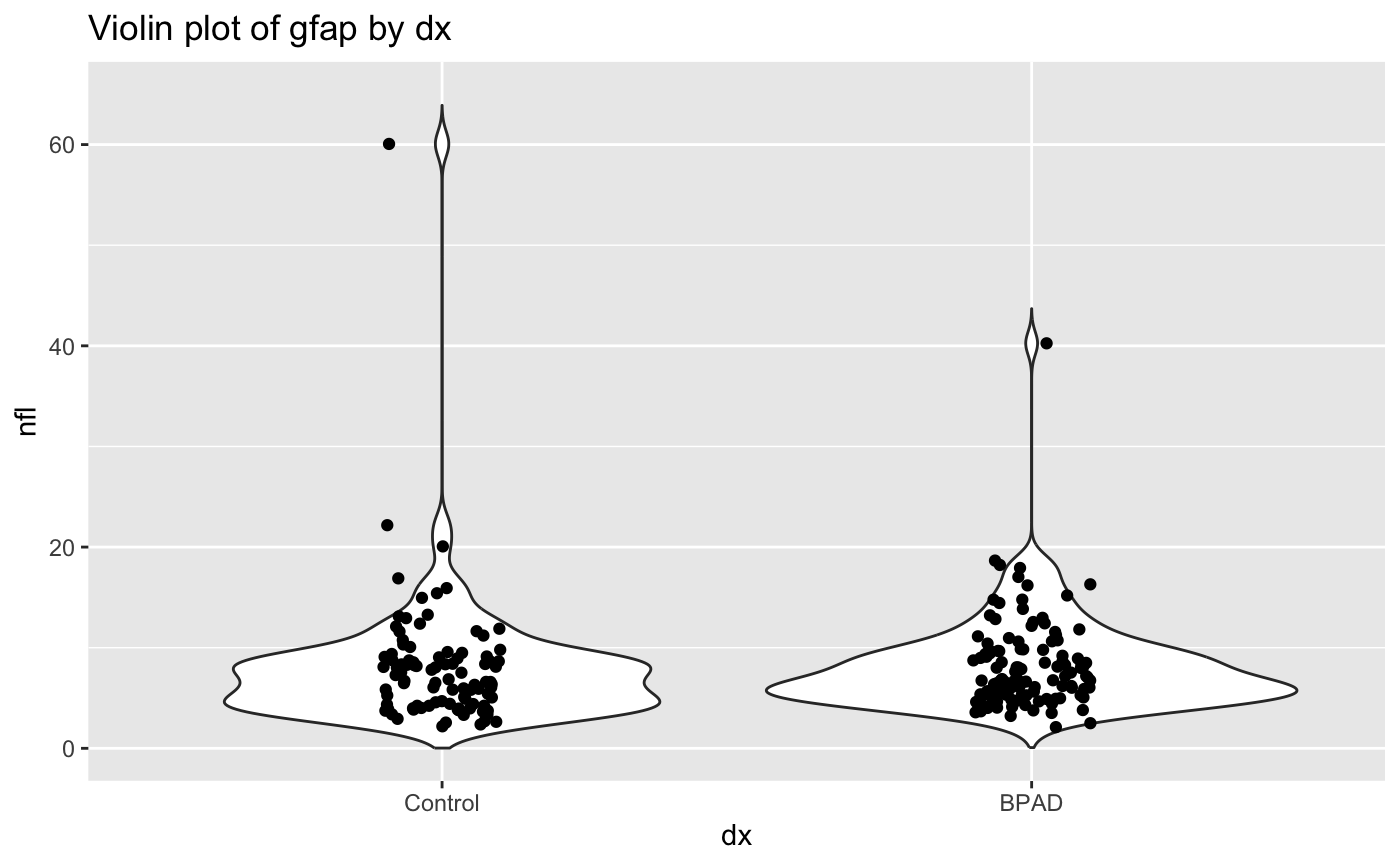


Diagnosis

NfL (pg/mL)

sFigure 1 – Violin and jitter plot of NfL in bipolar disorder and controls


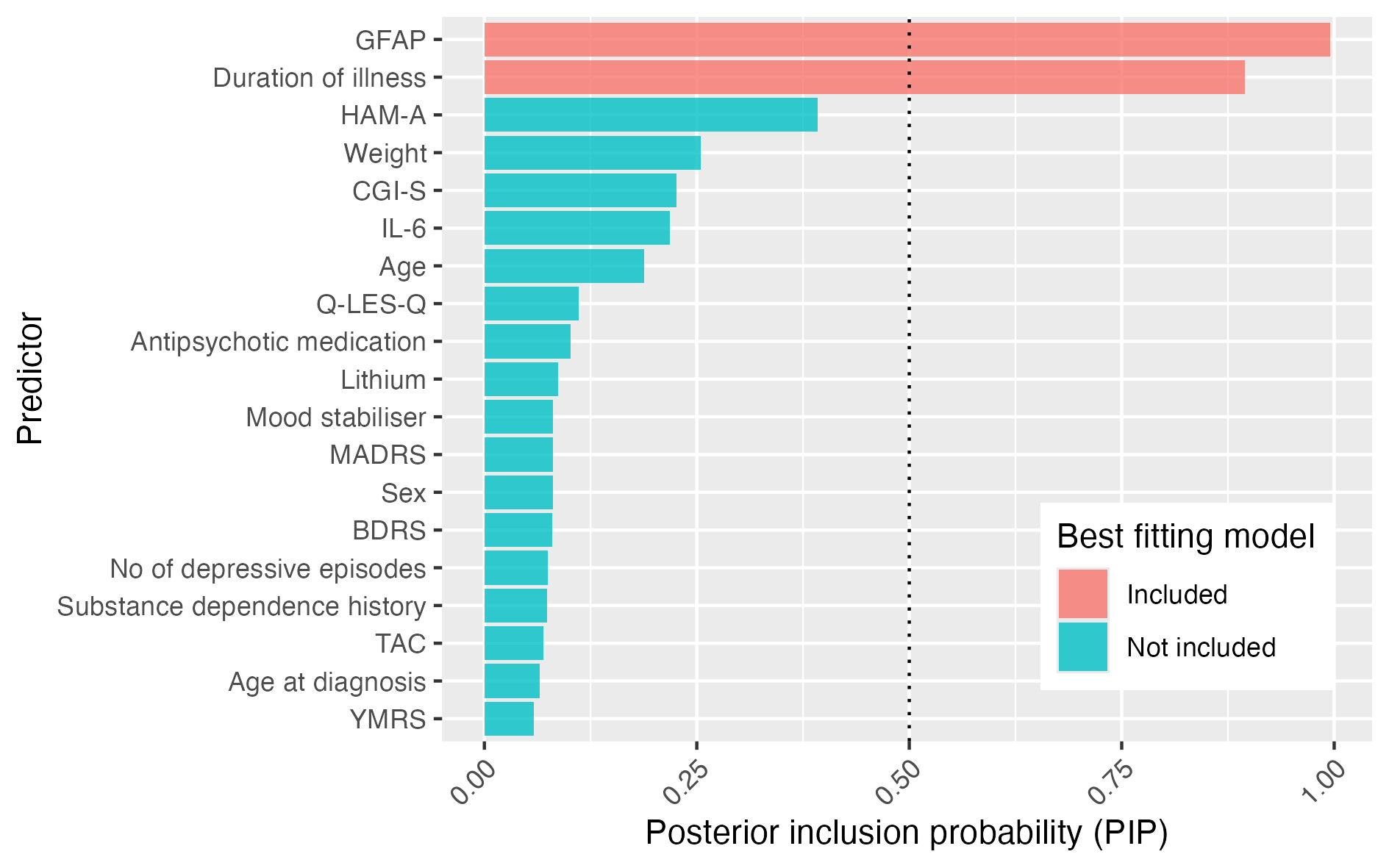


sFigure 2 - PIP for each predictor of NfL with averaged model parameters


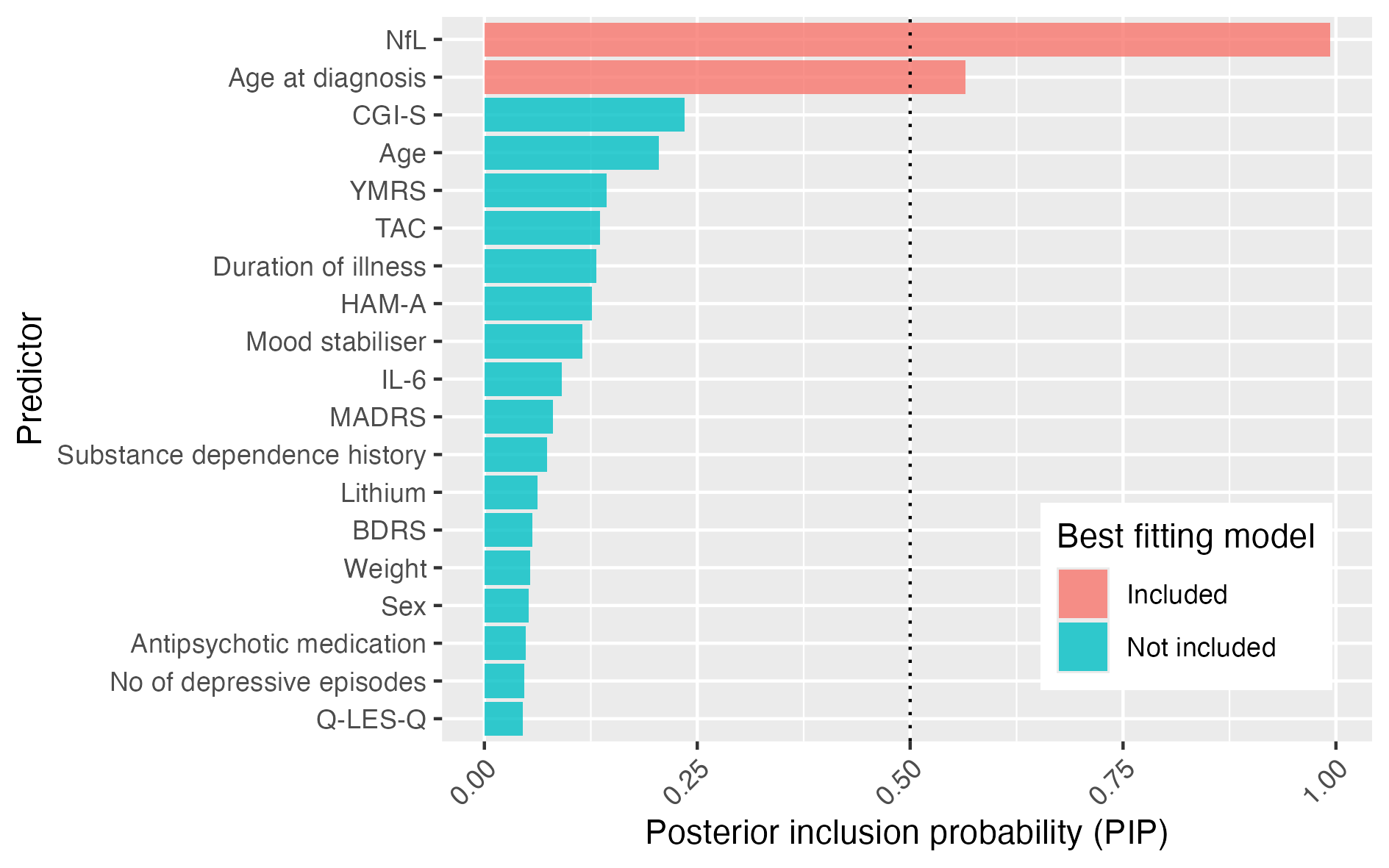


sFigure 3 - PIP for each predictor of GFAP with averaged model parameters

|  | NfL |  | GFAP |  |
| --- | --- | --- | --- | --- |
| Predictor | B [95% CI] | PIP | B [95% CI] | PIP |
| Age | 0.02 [0 – 0.15] | 0.19 | 0.22[0 – 1.59] | 0.21 |
| Sex | -0.06 [-1.00 – 0.04] | 0.08 | 0.27 [-0.02 – 0.86] | 0.05 |
| Weight | -0.01 [-0.06 – 0] | 0.26 | -0.01 [0 – 0.05] | 0.05 |
| Substance dependence history | -0.05 [-0.82 – 0] | 0.07 | -0.74 [-10.51 – 0] | 0.05 |
| Duration of illness | 0.13 [0 – 0.21] | 0.90 | -0.06 [-0.71 – 0] | 0.13 |
| Age at diagnosis | 0 [-0.01 – 0.02] | 0.06 | 0.67 [0 – 1.79] | 0.57 |
| Mood stabiliser | 0.06 [-0.01 – 0.89] | 0.08 | 1.49 [0 – 15.69] | 0.12 |
| Lithium | -0.09 [-1.27 – 0.06] | 0.09 | 0.64 [-1.60 – 2.09] | 0.06 |
| Antipsychotic | -0.09 [-1.17 – 0.01] | 0.10 | 0.20 [0 – 0] | 0.05 |
| No. depressive episodes | 0.03 [0 – 0.50] | 0.07 | 0.09 [0 – 0] | 0.05 |
| MADRS | 0 [-0.08 – 0.01] | 0.08 | 0.07 [0 – 1.12] | 0.08 |
| BDRS | 0 [-0.04 – 0.05] | 0.08 | 0.01 [-0.03 – 0.15] | 0.06 |
| HAM-A | -0.06 [-0.24 – 0] | 0.39 | 0.15 [0 – 1.55] | 0.12 |
| YMRS | 0 [-0.02 – 0.01] | 0.06 | -0.34 [-3.17 – 0.01] | 0.14 |
| Q-LES-Q | 0 [0 – 0.05] | 0.11 | 0 [0 – 0] | 0.05 |
| CGI-S | -0.23 [-1.57 – 0] | 0.23 | 2.68 [0 – 17.05] | 0.24 |
| IL-6 | 0.10 [0 – 0.71] | 0.22 | -0.17 [-2.61 – 0] | 0.09 |
| TAC | -0.15 [-2.69 - 0] | 0.07 | 8.04 [0 – 78.43] | 0.14 |
| GFAP | 0.04 [0.02 - 0.05] | 0.99 | N/A | N/A |
| NfL | N/A | N/A | 4.20 [2.17 – 6.30] | 0.99 |

sTable 1 - BMA analysis for prediction of plasma NfL and GFAP concentrations including number of depressive episodes.

B = model coefficients computed as the mean of the posterior distribution; SE = standard deviation of the posterior distributions; 95% CI = 95% credible intervals. PIP = posterior inclusion probability
